# Supplementary material for: Independent evolution of ancestral and novel defenses in a genus of toxic plants (Erysimum, Brassicaceae)
Source: eLife. 2020 Apr 7;9:e51712. doi: 10.7554/eLife.51712 (PMC7180059; doi:10.7554/eLife.51712)
Supplement: Supplementary file 1. [file elife-51712-supp1.docx]

**Supplementary File 1.** Origin of *Erysimum* species and seed material, and year of original collection where available. Four species from mislabeled seed stocks are referred to as accessions ER1-4, with false species name provided in quotation marks. Chromosome numbers and ploidy levels are inferred from chromosome counts in literature reports (reference provided in square brackets). For species with multiple ploidy levels, numbers are provided for sampled populations where known (highlighted by ^*^). Leaf material for each species or accession was collected in one of three experiments, and RNA was extracted from pooled tissue of 2-5 individual plants that were sampled at 1-2 time points (TP).

| Species code | Species | Region/country of origin | Seed origin | Original collection | Chromosomes (2n) | Ploidy | Source experiment | Pooled individuals |
| --- | --- | --- | --- | --- | --- | --- | --- | --- |
| ALI | *E. allionii*  (*E.* x. *marshallii*^A^) | n/a | Botanical Garden Kiel, Germany | 1985 | n/a | n/a | 2017-2 | 5 / 2TP |
| AMO | *E. amoenum* | Colorado, USA | Alplains, CO, USA | 2014 | 36^[^[^11^](#_ENREF_11)^]^ | 4x | 2017-1 | 5 / 1 TP |
| AND | *E. andrzejowskianum* | Austria | Collected | 2009 | 70^[^[^9^](#_ENREF_9)^]^ | 10x | 2017-1 | 5 / 2TP |
| BAE | *E. baeticum* | Spain | Collected | 2006 | 56^[^[^1^](#_ENREF_1)^]^ | 8x^*^ | 2016 | 5 / 1TP |
| BAS | *E. bastetanum* | Spain | Collected | 2007 | 42^[15]^ | 6x^*^ | 2017-2 | 5 / 2TP |
| BIC | *E. bicolor* | Canary Islands, Spain | Botanical Garden Konstanz, Germany | unknown | 28^[^[^6^](#_ENREF_6)^]^ | 4x | 2017-2 | 5 / 2TP |
| CAP | *E. capitatum* | USA | B&T World Seeds, France | unknown | 36^[^[^11^](#_ENREF_11)^]^ | 4x | 2017-1 | 4 / 2TP |
| CHR | *E. cheiri* | Netherlands | Collected | 2016 | 12^[^[^13^](#_ENREF_13)^]^ | 2x | 2017-2 | 5 / 2TP |
| COL | *E. collinum*^B^ | Iran | Botanical Garden Madrid, Spain | 1993 | 14^[^[^10^](#_ENREF_10)^]^ | 2x | 2017-1 | 3 / 1TP |
| CRA | *E. crassicaule* | Iran | Botanical Garden Madrid, Spain | unknown | 14^[^[^10^](#_ENREF_10)^]^ | 2x | 2017-2 | 2 / 2TP |
| CRE | *E. crepidifolium* | Czechia | Botanical Garden Berlin-Dahlem, Germany | 1996 | 14^[^[^13^](#_ENREF_13)^]^ | 2x | 2017-1 | 5 / 2TP |
| CSS | *E. crassipes* | Iran | Botanical Garden Madrid, Spain | 1993 | 14^[^[^10^](#_ENREF_10)^]^ | 2x | 2017-2 | 5 / 2TP |
| CUS | *E. cuspidatum* | Greece | Botanical Garden Berlin-Dahlem, Germany | 1980 | 16^[^[^10^](#_ENREF_10)^]^ | 2x | 2017-2 | 5 / 2TP |
| DIF | *E. diffusum* | Czechia | Botanical Garden Plzen, Czechia | 2013 | 28^[^[^13^](#_ENREF_13)^]^ | 4x | 2017-2 | 5 / 2TP |
| ECE | *E. cheiranthoides* | Germany | Collected | 2015 | 16^[^[^13^](#_ENREF_13)^]^ | 2x | 2017-1 | 5 / 1TP |
| ER1 | *Erysimum* sp. 1  *‘E. crepidifolium’* | n/a | Botanical Garden Berlin-Dahlem, Germany | unknown | n/a | n/a | 2017-1 | 4 / 1TP |
| ER2 | *Erysimum* sp. 2  *‘E. rhaeticum’* | n/a | Botanical Garden Nantes, France | unknown | n/a | n/a | 2017-2 | 5 / 2TP |
| ER3 | *Erysimum* sp. 3  ‘*E. asperum*’ | n/a | B&T World Seeds, France | unknown | n/a | n/a | 2017-1 | 5 / 2TP |
| ER4 | Erysimum sp. 4  ‘*E. suffrutescens*’ | n/a | B&T World Seeds, France | unknown | n/a | n/a | 2017-2 | 5 / 2TP |
| FIZ | *E. fitzii* | Spain | Collected | 2008 | 14^[^[^7^](#_ENREF_7)^]^ | 2x^*^ | 2017-2 | 2 / 2TP |
| FRA | *E. franciscanum* | California, USA | B&T World Seeds, France | unknown | 36^[^[^11^](#_ENREF_11)^]^ | 4x | 2017-1 | 5 / 2TP |
| HIE | *E. hieraciifolium* | Romania | Botanical Garden Jibou, Romania | unknown | 32^[^[^2^](#_ENREF_2)^]^ | 4x | 2017-2 | 5 / 2TP |
| HOR | *E. horizontale* | Greece | Botanical Garden Berlin-Dahlem, Germany | unknown | 24^[^[^10^](#_ENREF_10)^]^ | 4x | 2016 | 4 / 1TP |
| HUN | *E. hungaricum* | Romania | Botanical Garden Jibou, Romania | unknown | 48^[^[^9^](#_ENREF_9)^,^[^13^](#_ENREF_13)^]^ | 6x | 2017-2 | 5 / 2TP |
| INC | *E. incanum* | Spain | Botanical Garden Madrid, Spain | unknown | 16^[^[^7^](#_ENREF_7)^]^ | 2x | 2017-2 | 4 / 2TP |
| KOT | *E. kotschyanum* | Turkey | Botanical Garden Tübingen, Germany | unknown | 14^[^[^10^](#_ENREF_10)^]^ | 2x | 2017-2 | 5 / 2TP |
| LAG | *E. lagascae* | Spain | Botanical Garden Madrid, Spain | unknown | 14^[^[^7^](#_ENREF_7)^]^ | 2x^*^ | 2017-1 | 5 / 2TP |
| MAJ | *E. majellense* | Italy |  | unknown | 28^[^[^5^](#_ENREF_5)^]^ | 4x | 2016 | 5 / 1TP |
| MED | *E. mediohispanicum* | Spain | Collected | 2007 | 14^[^[^4^](#_ENREF_4)^]^ | 2x^*^ | 2017-2 | 5 / 2TP |
| MEX | *E. merxmuelleri* | Spain | Collected | 2007 | 14^[^[^7^](#_ENREF_7)^]^ | 2x^*^ | 2017-2 | 5 / 2TP |
| MEZ | *E. menziesii* | California, USA | B&T World Seeds, France | unknown | 36^[^[^3^](#_ENREF_3)^]^ | 4x | 2017-1 | 5 / 2TP |
| MIC | *E. microstylum* | Greece | Botanical Garden Berlin-Dahlem, Germany | 1980 | 14^[^[^8^](#_ENREF_8)^]^ | 2x | 2017-1 | 5 / 2TP |
| NAX | *E. naxense* | Greece | Balkan Botanic Garden of Korussia, Greece | unknown | 12^[^[^14^](#_ENREF_14)^]^ | 2x | 2016 | 5 / 1TP |
| NER | *E. nervosum* | Morocco | Collected | 2008 | 28^[^[^12^](#_ENREF_12)^]^ | 4x^*^ | 2017-1 | 5 / 1TP |
| NEV | *E. nevadense* | Spain | Collected | 2007 | 14^[^[^7^](#_ENREF_7)^]^ | 2x^*^ | 2017-2 | 3 / 2TP |
| ODO | *E. odoratum* | Austria | Botanical Garden Bern, Switzerland | 2015 | 32^[^[^13^](#_ENREF_13)^]^ | 4x | 2017-1 | 5 / 2TP |
| PIE | *E. pieninicum* | Romania | Botanical Garden Jibou, Romania | unknown | 48^[^[^13^](#_ENREF_13)^]^ | 6x | 2017-2 | 5 / 2TP |
| PSE | *E. pseudorhaeticum* | Italy | Botanical Garden Nantes, France | unknown | 14^[^[^5^](#_ENREF_5)^]^ | 2x | 2017-2 | 5 / 2TP |
| PUL | *E. pulchellum* | Turkey | Botanical Garden Koursk, Russia | unknown | 56^[^[^10^](#_ENREF_10)^]^ | 8x | 2017-2 | 5 / 2TP |
| REP | *E. repandum* | Spain | Botanical Garden Madrid, Spain | unknown | 16^[^[^7^](#_ENREF_7)^]^ | 2x | 2017-2 | 5 / 2TP |
| RHA | *E. rhaeticum* | Switzerland | Collected | 2016 | 56^[^[^13^](#_ENREF_13)^]^ | 8x | 2017-1 | 5 / 2TP |
| RUS | *E. ruscinonense* | Spain | Collected | 2007 | 14^[^[^7^](#_ENREF_7)^]^ | 2x | 2016 | 5 / 1TP |
| SCO | *E. scoparium* | Canary Islands, Spain | B&T World Seeds, France | unknown | 28^[^[^6^](#_ENREF_6)^]^ | 4x | 2017-1 | 4 / 1TP |
| SEM | *E. semperflorens* | Morocco | Collected | 2008 | 14^[^[^12^](#_ENREF_12)^]^ | 2x | 2017-1 | 5 / 2TP |
| SYL | *E. sylvestre* | Slovenia | B&T World Seeds, France | unknown | 14^[^[^13^](#_ENREF_13)^]^ | 2x | 2017-1 | 5 / 2TP |
| VIR | *E. virgatum* | Switzerland | Botanical Garden St Gallen, Switzerland | 1962 | 48^[^[^13^](#_ENREF_13)^]^ | 6x | 2017-2 | 5 / 2TP |
| WIC | *E. wilczekianum* | Morocco | Collected | 2008 | 16^[^[^12^](#_ENREF_12)^]^ | 4x^*^ | 2017-2 | 5 / 2TP |
| WIT | *E. witmannii* | Slovenia | Botanical Garden Berlin-Dahlem, Germany | 2010 | 14^[^[^13^](#_ENREF_13)^]^ | 2x | 2017-1 | 5 / 2TP |

^A^ horticultural hybrid; ^B^ synonymous for *E. passgalense* and *E. aucherianum*

**References**

1 Abdelaziz, M., Muñoz-Pajares, A. J., Lorite, J., Herrador, M. B., Perfectti, F. & Gómez, J. M. Phylogenetic relationships of *Erysimum* (Brassicaceae) from the Baetic Mountains (SE Iberian Peninsula). *Anales del Jardín Botánico de Madrid* **71**, e005, doi:10.3989/ajbm.2377 (2014).

2 Dvořák, F., Dadáková, B. & Grüll, F. Chromosome counts for *Erysimum durum*, *Erysimum hieraciifolium* and *Chenopodium album*. *Folia Geobotanica et Phytotaxonomica* **10**, 185-190, doi:10.1007/bf02852860 (1975).

3 Flora of North America Editorial Committee. Flora of North America: Volume 7: Magnoliophyta: Dilleniidae, Part 2 (2010). Oxford University Press.

4 Muñoz-Pajares, A. J., Perfectti, F., Loureiro, J., Abdelaziz, M., Biella, P., Castro, M., Castro, S. & Gómez, J. M. Niche differences may explain the geographic distribution of cytotypes in *Erysimum mediohispanicum*. *Plant Biology* **20**, 139-147, doi:10.1111/plb.12605 (2018).

5 Peccenini, S. The genus *Erysimum* (Brassicaceae) in Italy, part I. *Annalen des Naturhistorischen Museums in Wien – Serie B* **114**, 95-128 (2012).

6 Polatschek, A. Die Gattung *Erysimum* auf den Kapverden, Kanaren und Madeira. *Annalen des Naturhistorischen Museums in Wien* **80**, 93-103 (1976).

7 Polatschek, A. Die Arten der Gattung Erysimum auf der Iberischen Halbinsel. *Annalen des Naturhistorischen Museums in Wien* **82**, 325-362 (1979).

8 Polatschek, A. *Erysimum* (Brassicaceae): Chromosomenzählungen griechischer Arten *Linzer Biologischer Beiträge* **29**, 545-553 (1997).

9 Polatschek, A. Revision der Gattung *Erysimum* (Cruciferae): Teil 1: Russland, die Nachfolgestaaten der USSR (excl. Georgien, Armenien, Azerbaidzan), China, Indien, Pakistan, Japan und Korea. *Annalen des Naturhistorischen Museums in Wien – Serie B* **111** (2010).

10 Polatschek, A. Revision der Gattung *Erysimum* (Cruciferae), Teil 2: Georgien, Armenien, Azerbaidzan, Türkei, Syrien, Libanon, Israel, Jordanien, Irak, Iran, Afghanistan. *Annalen des Naturhistorischen Museums in Wien – Serie B* **112** (2011).

11 Polatschek, A. Revision der Gattung *Erysimum* (Cruciferae), Teil 3: Amerika und Grönland. . *Annalen des Naturhistorischen Museums in Wien – Serie B* **113**, 139-192 (2012).

12 Polatschek, A. Revision der Gattung Erysimum (Cruciferae), Teil 4: Nordafrika, Malta und Zypern. *Annalen des Naturhistorischen Museums in Wien – Serie B* **115**, 57-74 (2013).

13 Polatschek, A. Revision der Gattung *Erysimum* (Cruciferae), Teil 5: Nord-, West-, Zentraleuropa, Rumänien und westliche Balkan-Halbinsel bis Albanien. *Annalen des Naturhistorischen Museums in Wien – Serie B* **115**, 75-218 (2013).

14 Snogerup, S. Studies in the Aegean flora. VIII. *Erysimum* sect. *Cheiranthus*. A. Taxonomy. *Opera Botanica* **13**, 1-70 (1967).

15 Perfectti, F. & Gomez, J. M. personal communication
