# Supplementary material for: Independent evolution of ancestral and novel defenses in a genus of toxic plants (Erysimum, Brassicaceae)
Source: eLife. 2020 Apr 7;9:e51712. doi: 10.7554/eLife.51712 (PMC7180059; doi:10.7554/eLife.51712)
Supplement: Supplementary file 2. [file elife-51712-supp2.docx]

**Supplementary File 2.** Repetitive sequences and transposable elements in the *E. cheiranthoides* genome.

| Classification | Order | Superfamily | No. of TEs | Coverage (Mb) | Fraction of genome (%) |
| --- | --- | --- | --- | --- | --- |
| Class I | LTR | *Copia* | 3508 | 2.643 | 1.492 |
|  | LTR | *Gypsy* | 13748 | 13.371 | 7.547 |
|  | LTR | Unknown/Other | 24145 | 11.96 | 6.75 |
|  | LINE | *L1* | 6105 | 2.585 | 1.459 |
| Class II | TIR | *CMC-EnSpm* | 1222 | 0.543 | 0.307 |
|  | TIR | *hAT* | 1225 | 0.42 | 0.237 |
|  | Helitron | *Helitron* | 1292 | 0.763 | 0.431 |
|  | TIR | *Mutator* | 2880 | 1.406 | 0.794 |
|  | TIR | PIF-Harbinger | 569 | 0.227 | 0.128 |
|  |  | Unknown | 27 | 0.002 | 0.001 |
| Other Simple Repeats |  |  | 641 | 0.124 | 0.07 |
| Other Unknown Repeats |  |  | 45324 | 16.618 | 9.379 |
| **Total** |  |  |  |  | **28.59** |
