# Supplementary material for: Independent evolution of ancestral and novel defenses in a genus of toxic plants (Erysimum, Brassicaceae)
Source: eLife. 2020 Apr 7;9:e51712. doi: 10.7554/eLife.51712 (PMC7180059; doi:10.7554/eLife.51712)
Supplement: Supplementary file 3. [file elife-51712-supp3.docx]

**Supplementary File 3.** Transcriptome assembly metrics, including number of sequences, N50 values, and recovered BUSCO gene number. Additionally, transcript lengths were divided by the length of the top BLAST match to the *E. cheiranthoides* v1.1 gene model (EC1.1) to determine fragmentation of the transcriptome assemblies (tophit average trinity_len/EC_len). RNA sequences from each of the 48 *Erysimum* species were mapped to the *E. cheiranthoides* genome, and results are reported as the number of E. cheiranthoides genes represented and the mapping percentage.

| Species | Sequence count | N50  [bp] | BUSCO genes | | | | | Tophit average trinity_len/EC_len | No. of EC1.1 genes represented | Mapping to EC1.1 (%) |
| --- | --- | --- | --- | --- | --- | --- | --- | --- | --- | --- |
|  |  |  | Complete (percent total) | Complete single-copy | Complete duplicated | Fragmented | Missing |  |  |  |
| ALI | 207422 | 595 | 1010 (70.1%) | 276 | 734 | 250 | 180 | 0.74 | 19150 | 56.6 |
| AMO | 217430 | 888 | 903 (62.7%) | 419 | 484 | 323 | 214 | 0.97 | 21450 | 59.4 |
| AND | 165687 | 1172 | 1043 (72.4%) | 445 | 598 | 252 | 145 | 1.09 | 20995 | 58.5 |
| AUC | 164506 | 1367 | 1163 (80.8%) | 439 | 724 | 183 | 94 | 1.21 | 20711 | 58.6 |
| BAE | 180487 | 1127 | 1041 (72.3%) | 479 | 562 | 253 | 146 | 1.05 | 21187 | 59.1 |
| BAS | 135035 | 1420 | 1183 (82.2%) | 442 | 741 | 147 | 110 | 0.91 | 20279 | 57.8 |
| BIC | 99234 | 1868 | 1314 (91.3%) | 519 | 795 | 67 | 59 | 1.13 | 19925 | 57.8 |
| CAP | 260998 | 842 | 887 (61.6%) | 406 | 481 | 366 | 187 | 0.97 | 22113 | 57.4 |
| CHR | 93525 | 1963 | 1350 (93.8%) | 565 | 785 | 39 | 51 | 1.22 | 20213 | 57.3 |
| CRA | 143666 | 1582 | 1266 (87.9%) | 417 | 849 | 113 | 61 | 1.01 | 20924 | 54.2 |
| CRE | 102472 | 1768 | 1321 (91.7%) | 589 | 732 | 55 | 64 | 1.28 | 19686 | 59 |
| CSS | 118038 | 1588 | 1241 (86.2%) | 469 | 773 | 90 | 108 | 1.01 | 20457 | 56.4 |
| CUS | 122476 | 1644 | 1263 (87.7%) | 457 | 806 | 110 | 67 | 1.02 | 20077 | 58.1 |
| DIF | 139288 | 770 | 1189 (82.6%) | 392 | 797 | 154 | 97 | 0.89 | 20472 | 56.7 |
| ECE | 81984 | 2139 | 1341 (93.1%) | 658 | 683 | 34 | 65 | 1.41 | 19519 | 94.8 |
| ER1 | 223508 | 900 | 951 (66.0%) | 378 | 573 | 287 | 202 | 1.01 | 21335 | 66.5 |
| ER2 | 258578 | 715 | 785 (54.5%) | 307 | 478 | 379 | 276 | 0.57 | 23080 | 55.1 |
| ER3 | 123653 | 1886 | 1338 (92.9%) | 452 | 886 | 40 | 62 | 1.35 | 20130 | 77.5 |
| ER4 | 89871 | 1844 | 1328 (92.2%) | 469 | 859 | 49 | 63 | 1.13 | 23371 | 78.2 |
| FIZ | 94064 | 1956 | 1320 (91.7%) | 551 | 769 | 50 | 70 | 1.21 | 21130 | 59.1 |
| FRA | 220291 | 1004 | 992 (68.9%) | 384 | 608 | 284 | 164 | 1.04 | 21412 | 59.9 |
| HIE | 218565 | 955 | 960 (66.7%) | 296 | 664 | 286 | 194 | 0.71 | 20878 | 66 |
| HOR | 109045 | 1784 | 1306 (90.7%) | 501 | 805 | 77 | 57 | 1.29 | 19758 | 61.9 |
| HUN | 228679 | 881 | 903 (62.7%) | 291 | 612 | 313 | 224 | 0.67 | 21558 | 61.4 |
| INC | 90295 | 2002 | 1337 (92.8%) | 481 | 856 | 40 | 63 | 1.23 | 20671 | 61.4 |
| KOT | 104476 | 1755 | 1322 (91.8%) | 531 | 791 | 56 | 62 | 1.07 | 21558 | 56.8 |
| LAG | 155771 | 1422 | 1196 (83.1%) | 419 | 777 | 153 | 91 | 1.21 | 20777 | 59.1 |
| MAJ | 174858 | 1027 | 958 (66.5%) | 462 | 496 | 286 | 196 | 1.01 | 20830 | 57.9 |
| MED | 106039 | 1869 | 1301 (90.3%) | 506 | 795 | 69 | 70 | 1.41 | 20907 | 57.9 |
| MEX | 102500 | 1115 | 1284 (89.2%) | 501 | 783 | 74 | 82 | 1.13 | 21693 | 57.4 |
| MEZ | 187227 | 1160 | 1074 (74.6%) | 420 | 654 | 203 | 163 | 1.1 | 20811 | 56.9 |
| MIC | 115738 | 1696 | 1271 (88.3%) | 513 | 758 | 94 | 75 | 1.29 | 19993 | 61.2 |
| NAX | 104553 | 2016 | 1351 (93.8%) | 549 | 802 | 32 | 57 | 1.37 | 19589 | 60.5 |
| NER | 161135 | 1485 | 1228 (85.3%) | 471 | 757 | 133 | 79 | 1.23 | 20833 | 59.4 |
| NEV | 115096 | 1710 | 1282 (89.0%) | 450 | 832 | 84 | 74 | 1.08 | 19160 | 59.6 |
| ODO | 193451 | 1087 | 975 (67.7%) | 421 | 554 | 289 | 176 | 1.07 | 21108 | 57.9 |
| PIE | 238425 | 878 | 887 (61.6%) | 275 | 612 | 327 | 226 | 0.69 | 22492 | 59.5 |
| PSE | 129384 | 1513 | 1230 (85.4%) | 428 | 802 | 134 | 76 | 0.95 | 22784 | 57.4 |
| PUL | 251705 | 741 | 773 (53.7%) | 305 | 468 | 391 | 276 | 0.58 | 20633 | 54.6 |
| REP | 64315 | 2160 | 1340 (93.1%) | 727 | 613 | 34 | 66 | 1.33 | 22932 | 57.3 |
| RHA | 220584 | 876 | 887 (61.6%) | 440 | 447 | 335 | 218 | 0.97 | 21715 | 57.4 |
| RUS | 154276 | 1328 | 1125 (78.1%) | 470 | 655 | 188 | 127 | 1.13 | 20616 | 60.7 |
| SCO | 92945 | 1897 | 1331 (92.4%) | 625 | 706 | 48 | 61 | 1.34 | 19314 | 61.2 |
| SEM | 91502 | 1875 | 1317 (91.5%) | 608 | 709 | 51 | 72 | 1.34 | 19479 | 59.9 |
| SYL | 113269 | 1918 | 1331 (92.4%) | 449 | 882 | 44 | 65 | 1.37 | 20013 | 77.8 |
| VIR | 212293 | 574 | 990 (68.8%) | 305 | 685 | 278 | 172 | 0.72 | 22901 | 66.1 |
| WIC | 91007 | 2039 | 1339 (93.0%) | 586 | 753 | 36 | 65 | 1.28 | 23344 | 56.9 |
| WTT | 171465 | 1768 | 1284 (89.2%) | 395 | 889 | 97 | 59 | 1.37 | 21116 | 57.8 |
