# Supplementary material for: Independent evolution of ancestral and novel defenses in a genus of toxic plants (Erysimum, Brassicaceae)
Source: eLife. 2020 Apr 7;9:e51712. doi: 10.7554/eLife.51712 (PMC7180059; doi:10.7554/eLife.51712)
Supplement: Supplementary file 4. [file elife-51712-supp4.docx]

**Supplementary File 4.** Discordance metrics for the ASTRAL phylogeny. Node numbers correspond to the labels on the ASTRAL species tree (Figure 5-figure supplement 1). q1, q2, q3: quartet support for the main topology (presented), the first alternative, and the second alternative, respectively. f1, f2, f3: total number of quartet trees in all the gene trees that support the main topology, the first alternative, and the second alternative, respectively. pp1, pp2, pp3: local posterior probabilities for the main topology, the first alternative, and the second alternative, respectively.

| **Node** | **q1** | **q2** | **q3** | **f1** | **f2** | **f3** | **pp1** | **pp2** | **pp3** |
| --- | --- | --- | --- | --- | --- | --- | --- | --- | --- |
| 1 | 0.39 | 0.29 | 0.32 | 3133 | 2357 | 2525 | 1.00 | 0.00 | 0.00 |
| 2 | 0.37 | 0.32 | 0.31 | 3184 | 2780 | 2204 | 1.00 | 0.00 | 0.00 |
| 3 | 0.38 | 0.32 | 0.31 | 3222 | 2763 | 2548 | 1.00 | 0.00 | 0.00 |
| 4 | 0.35 | 0.34 | 0.32 | 2952 | 2864 | 2687 | 0.97 | 0.02 | 0.00 |
| 5 | 0.35 | 0.33 | 0.32 | 3054 | 2842 | 2752 | 1.00 | 0.00 | 0.00 |
| 6 | 0.35 | 0.31 | 0.33 | 3011 | 2683 | 2851 | 1.00 | 0.00 | 0.00 |
| 7 | 0.35 | 0.34 | 0.31 | 3006 | 2939 | 2704 | 0.96 | 0.03 | 0.00 |
| 8 | 0.35 | 0.34 | 0.30 | 3255 | 3150 | 2800 | 1.00 | 0.00 | 0.00 |
| 9 | 0.37 | 0.32 | 0.30 | 3425 | 2985 | 2798 | 1.00 | 0.00 | 0.00 |
| 10 | 0.57 | 0.21 | 0.22 | 3878 | 1826 | 1850 | 1.00 | 0.00 | 0.00 |
| 11 | 0.52 | 0.25 | 0.23 | 3850 | 2302 | 2165 | 1.00 | 0.00 | 0.00 |
| 12 | 0.34 | 0.33 | 0.33 | 3138 | 3111 | 3088 | 0.50 | 0.29 | 0.20 |
| 13 | 0.43 | 0.29 | 0.28 | 3989 | 2739 | 2631 | 1.00 | 0.00 | 0.00 |
| 14 | 0.34 | 0.33 | 0.32 | 3025 | 2941 | 2866 | 0.89 | 0.08 | 0.03 |
| 15 | 0.35 | 0.32 | 0.33 | 2938 | 2735 | 2747 | 1.00 | 0.00 | 0.00 |
| 16 | 0.37 | 0.32 | 0.31 | 3437 | 3005 | 2886 | 1.00 | 0.00 | 0.00 |
| 17 | 0.36 | 0.31 | 0.33 | 3531 | 3087 | 3237 | 1.00 | 0.00 | 0.00 |
| 18 | 0.39 | 0.30 | 0.31 | 3617 | 2752 | 2879 | 1.00 | 0.00 | 0.00 |
| 19 | 0.44 | 0.30 | 0.26 | 3566 | 2395 | 2137 | 1.00 | 0.00 | 0.00 |
| 20 | 0.35 | 0.33 | 0.32 | 3475 | 3213 | 3179 | 1.00 | 0.00 | 0.00 |
| 21 | 0.36 | 0.31 | 0.33 | 3526 | 3045 | 3295 | 1.00 | 0.00 | 0.00 |
| 22 | 0.35 | 0.35 | 0.30 | 3189 | 3174 | 2743 | 0.74 | 0.26 | 0.00 |
| 23 | 0.35 | 0.33 | 0.31 | 3212 | 3016 | 2826 | 1.00 | 0.00 | 0.00 |
| 24 | 0.34 | 0.34 | 0.32 | 3113 | 3037 | 2879 | 0.93 | 0.05 | 0.01 |
| 25 | 0.37 | 0.32 | 0.31 | 3521 | 3113 | 2983 | 1.00 | 0.00 | 0.00 |
| 26 | 0.59 | 0.20 | 0.20 | 5159 | 1757 | 1763 | 1.00 | 0.00 | 0.00 |
| 27 | 0.45 | 0.27 | 0.27 | 3937 | 2362 | 2364 | 1.00 | 0.00 | 0.00 |
| 28 | 0.36 | 0.32 | 0.32 | 3548 | 3153 | 3019 | 1.00 | 0.00 | 0.00 |
| 29 | 0.41 | 0.30 | 0.29 | 3463 | 2560 | 2500 | 1.00 | 0.00 | 0.00 |
| 30 | 0.36 | 0.31 | 0.33 | 3463 | 3033 | 3216 | 1.00 | 0.00 | 0.00 |
| 31 | 0.35 | 0.32 | 0.33 | 3282 | 2923 | 3058 | 1.00 | 0.00 | 0.00 |
| 32 | 0.48 | 0.26 | 0.25 | 4133 | 2260 | 2178 | 1.00 | 0.00 | 0.00 |
| 33 | 0.48 | 0.26 | 0.27 | 4151 | 2237 | 2324 | 1.00 | 0.00 | 0.00 |
| 34 | 0.36 | 0.34 | 0.30 | 3577 | 3316 | 2973 | 1.00 | 0.00 | 0.00 |
| 35 | 0.37 | 0.33 | 0.29 | 3641 | 3277 | 2873 | 1.00 | 0.00 | 0.00 |
| 36 | 0.40 | 0.30 | 0.30 | 3589 | 2739 | 2692 | 1.00 | 0.00 | 0.00 |
| 37 | 0.35 | 0.34 | 0.30 | 3331 | 3206 | 2856 | 1.00 | 0.00 | 0.00 |
| 38 | 0.35 | 0.33 | 0.32 | 3092 | 2851 | 2806 | 1.00 | 0.00 | 0.00 |
| 39 | 0.40 | 0.30 | 0.30 | 3940 | 2971 | 2902 | 1.00 | 0.00 | 0.00 |
| 40 | 0.43 | 0.33 | 0.24 | 4116 | 3208 | 2360 | 1.00 | 0.00 | 0.00 |
| 41 | 0.51 | 0.19 | 0.31 | 4225 | 1562 | 2543 | 1.00 | 0.00 | 0.00 |
| 42 | 0.50 | 0.38 | 0.12 | 4392 | 3325 | 1052 | 1.00 | 0.00 | 0.00 |
| 43 | 0.42 | 0.32 | 0.26 | 3487 | 2660 | 2107 | 1.00 | 0.00 | 0.00 |
| 44 | 0.35 | 0.34 | 0.31 | 2824 | 2781 | 2645 | 0.89 | 0.11 | 0.00 |
| 45 | 0.42 | 0.31 | 0.27 | 3675 | 2749 | 2379 | 1.00 | 0.00 | 0.00 |
| 46 | 0.60 | 0.21 | 0.19 | 4495 | 1606 | 1417 | 1.00 | 0.00 | 0.00 |
