# Supplementary material for: Independent evolution of ancestral and novel defenses in a genus of toxic plants (Erysimum, Brassicaceae)
Source: eLife. 2020 Apr 7;9:e51712. doi: 10.7554/eLife.51712 (PMC7180059; doi:10.7554/eLife.51712)
Supplement: Supplementary file 5. [file elife-51712-supp5.docx]

**Supplementary File 5.** Discordance metrics for the ExaML phylogeny. Node numbers correspond to the labels on the ExaML species tree (Figure 5), while Treefile_node corresponds to node numbers in the ExaML tree file (provided on the Dryad repository). gCF are gene concordance factors and represent the percentage of gene trees that agree with the main topology at each node. gDF1 and gDF2 are discordance factors in percent for NNI-1 and NNI-2 branches, and gN are the numbers of trees decisive for each branch. Length is the branch length.

| Node | Treefile_Node | gCF | gDF1 | gDF2 | gN | Length |
| --- | --- | --- | --- | --- | --- | --- |
| 1 | 78 | 12.85 | 2.41 | 2.22 | 8389 | 0.004306 |
| 2 | 77 | 3.23 | 0.57 | 0.44 | 9545 | 0.003539 |
| 3 | 79 | 5.86 | 4.57 | 3.43 | 8670 | 0.003419 |
| 4 | 76 | 0.52 | 0.86 | 0.43 | 9694 | 0.002073 |
| 5 | 80 | 6.03 | 1.03 | 0.48 | 8506 | 0.003588 |
| 6 | 74 | 0.47 | 0.26 | 0.15 | 9778 | 0.002263 |
| 7 | 75 | 4.25 | 0.38 | 0.18 | 8743 | 0.003873 |
| 8 | 71 | 1.52 | 0.92 | 0.29 | 9300 | 0.002821 |
| 9 | 72 | 2.34 | 0.34 | 0.44 | 9014 | 0.00231 |
| 10 | 70 | 3.67 | 0.04 | 0 | 9322 | 0.007139 |
| 11 | 73 | 29.42 | 2.76 | 2.56 | 8012 | 0.007794 |
| 12 | 69 | 0.11 | 0.06 | 0.02 | 9857 | 0.003045 |
| 13 | 83 | 7.49 | 3.4 | 3.95 | 8811 | 0.004448 |
| 14 | 82 | 2.94 | 0.74 | 0.97 | 9248 | 0.005682 |
| 15 | 84 | 8.3 | 3.09 | 3.59 | 8829 | 0.005379 |
| 16 | 81 | 0.61 | 0.64 | 0.17 | 9338 | 0.003975 |
| 17 | 66 | 0.03 | 0 | 0 | 9857 | 0.002818 |
| 18 | 68 | 15.46 | 3.81 | 5.38 | 8100 | 0.006045 |
| 19 | 67 | 3.47 | 0.02 | 0.01 | 9250 | 0.005224 |
| 20 | 96 | 18.15 | 3.92 | 3.63 | 8573 | 0.007055 |
| 21 | 95 | 3.11 | 0.62 | 0.62 | 9259 | 0.003539 |
| 22 | 65 | 0.01 | 0 | 0.01 | 9868 | 0.002317 |
| 23 | 94 | 0.22 | 0.53 | 0.4 | 9714 | 0.002415 |
| 24 | 97 | 9.26 | 0.46 | 0.25 | 8520 | 0.006982 |
| 25 | 92 | 0.11 | 0 | 0 | 9793 | 0.00352 |
| 26 | 93 | 16.49 | 0.03 | 0.09 | 8715 | 0.009689 |
| 27 | 85 | 0 | 0 | 0 | 9868 | 0.002769 |
| 28 | 90 | 6.39 | 5.17 | 4.78 | 8750 | 0.005309 |
| 29 | 89 | 1.59 | 1.09 | 0.85 | 9395 | 0.004273 |
| 30 | 60 | 0.05 | 0 | 0 | 9868 | 0.003202 |
| 31 | 88 | 0.44 | 0.03 | 0.1 | 9738 | 0.004777 |
| 32 | 91 | 10.76 | 0.52 | 0.44 | 9013 | 0.008966 |
| 33 | 86 | 0 | 0.01 | 0 | 9811 | 0.003347 |
| 34 | 87 | 4.91 | 0.04 | 0 | 9031 | 0.007565 |
| 35 | 63 | 29.38 | 0.76 | 0.63 | 8680 | 0.012084 |
| 36 | 62 | 1.13 | 0.89 | 1.21 | 8948 | 0.003526 |
| 37 | 54 | 2.84 | 0.1 | 0.09 | 9814 | 0.004362 |
| 38 | 64 | 13.23 | 1.63 | 1.51 | 8664 | 0.007331 |
| 39 | 61 | 0.15 | 0.01 | 0.01 | 9106 | 0.002629 |
| 40 | 59 | 14.48 | 14.78 | 10.86 | 7962 | 0.005761 |
| 41 | 58 | 9.6 | 15.52 | 6.88 | 8478 | 0.004393 |
| 42 | 57 | 10.79 | 4.45 | 0.37 | 9015 | 0.013031 |
| 43 | 51 | 100 | 0 | 0 | 9814 | 0.065477 |
| 44 | 55 | 1.2 | 0.31 | 0.05 | 9686 | 0.006541 |
| 45 | 56 | 18.98 | 0.25 | 1.15 | 8332 | 0.009334 |
| 46 | 53 | 35.29 | 5.47 | 3.96 | 7520 | 0.009465 |
| 47 | 52 | 8.8 | 3.91 | 4.71 | 8798 | 0.004602 |
