# Supplementary material for: Independent evolution of ancestral and novel defenses in a genus of toxic plants (Erysimum, Brassicaceae)
Source: eLife. 2020 Apr 7;9:e51712. doi: 10.7554/eLife.51712 (PMC7180059; doi:10.7554/eLife.51712)
Supplement: Supplementary file 6. [file elife-51712-supp6.docx]

**Supplementary File 6.** List of glucosinolate compounds, determined by exact mass, fragmentation patterns, and retention time. Asterisks (*) indicate compounds confirmed by commercial standards.

| # | Systematic short name | Systematic name  (- glucosinolate) | Common name | Class | Molecular formula | Retention time | [M-H]^-^ | MS fragments |
| --- | --- | --- | --- | --- | --- | --- | --- | --- |
| 1 | 3MTP | 3-methylthiopropyl | Glucoiberverin | Aliphatic | C_11_H_21_NO_9_S_3_ | 3.16 | 406.0300 | 259.013, 241.001, 195.033, 96.960 |
| 2 | 3MSI | 3-methylsulfinylpropyl* | Glucoiberin | Aliphatic | C_11_H_21_NO_10_S_3_ | 1.86 | 422.0249 | 358.0276^1^, 259.014, 195.034, 96.961 |
| 3 | 2OH | 2-hydroxypropyl | - | Aliphatic | C_10_H_19_NO_10_S_2_ | 1.76 | 376.0372 | 259.013, 195.034, 96.960 |
| 4 | 2PRO | 2-propenyl* | Sinigrin | Aliphatic | C_10_H_17_NO_9_S_2_ | 2.33 | 358.0266 | 259.013, 241.003, 195.033, 96.960 |
| 5 | 3MSO | 3-methylsulfonylpropyl* | Glucocheirolin | Aliphatic | C_11_H_21_NO_11_S_3_ | 2.06 | 438.0198 | 259.013, 241.002, 195.033, 96.960 |
| 6 | 3MSO’ | *3-methylsulfonylpropyl isomer* |  | Aliphatic | C_11_H_21_NO_11_S_3_ | 1.75 | 438.0202 | 96.961 |
| 7 | 1MP | 1-methylpropyl | - | Aliphatic | C_11_H_21_NO_9_S_2_ | 3.13 | 374.0579 | 96.961 |
| 8 | 2MP | 2-methylpropyl | - | Aliphatic | C_11_H_21_NO_9_S_2_ | 3.21 | 374.0579 | 96.960 |
| 9 | 4MTB | 4-methylthiobutyl | Glucoerucin | Aliphatic | C_12_H_23_NO_9_S_3_ | 3.69 | 420.0456 | 96.960 |
| 10 | 4MSI | 4-methylsulfinylbutyl | Glucoraphanin | Aliphatic | C_12_H_23_NO_10_S_3_ | 2.03 | 436.0405 | 372.043^1^, 259.013, 195.034, 96.961 |
| 11 | 4BUT | 3-butenyl | Gluconapin | Aliphatic | C_11_H_19_NO_9_S_2_ | 2.90 | 372.0423 | 96.960 |
| 12 | NMB | n-methylbutyl | - | Aliphatic | C_12_H_23_NO_9_S_2_ | 3.96 | 388.0736 | 96.960 |
| 13 | 4MSO | 4-methylsulfonylbutyl | Glucoerysolin | Aliphatic | C_12_H_23_NO_11_S_3_ | 2.24 | 452.0355 | 96.961 |
| 14 | OH4MSO | 3-hydroxy-4-methylsulfonylbutyl | - | Aliphatic | C_12_H_23_NO_12_S_3_ | 1.99 | 468.0304 | 259.013, 195.033, 96.961 |
| 15 | 5MTP | 5-methylthiopentyl | Glucoberteroin | Aliphatic | C_13_H_25_NO_9_S_3_ | 4.31 | 434.0613 | 96.961 |
| 16 | 5MSI | 5-methylsulfinylpentyl | Glucoalyssin | Aliphatic | C_13_H_25_NO_10_S_3_ | 2.32 | 450.0562 | 386.059^1^, 259.013, 96.961 |
| 17 | 5MSO | 5-methylsulfonylpentyl | - | Aliphatic | C_13_H_25_NO_11_S_3_ | 2.55 | 466.0511 | 259.013, 241.004, 195.033, 96.961 |
| 18 | OH5MSO | 3-hydroxy-5-methylsulfonylpentyl | - | Aliphatic | C_13_H_25_NO_12_S_3_ | 2.11 | 482.0460 | 259.013, 195.033, 96.961 |
| 19 | 6MSI | 6-methylsulfinylhexyl | Glucohesperin | Aliphatic | C_14_H_27_NO_10_S_3_ | 2.69 | 464.0719 | 400.075^1^, 259.013. 96.960 |
| 20 | 6MSO | 6-methylsulfonylhexyl | - | Aliphatic | C_14_H_27_NO_11_S_3_ | 2.99 | 480.0668 | 259.013, 195.033,96.960 |
| 21 | OH6MSO | 3-hydroxy-6-methylsulfonylhexyl | - | Aliphatic | C_14_H_27_NO_12_S_3_ | 2.31 | 496.0617 | 259.013, 96.960 |
| 22 | 3MECOP | 3-methoxycarbonylpropyl | Glucoerypestrin | Carboxylic | C_12_H_21_NO_11_S_2_ | 2.75 | 418.0477 | 259.013, 195.033, 96.961 |
| 23 | I3M | indol-3-ylmethyl | Glucobrassicin | Indole | C_16_H_20_N_2_O_9_S_2_ | 4.00 | 447.0532 | 96.960 |
| 24 | 4OHI3M | 4-hydroxy-indol-3-ylmethyl | 4-Hydroxyglucobrassicin | Indole | C_16_H_20_N_2_O_10_S_2_ | 3.37 | 463.0481 | 259.013, 96.961 |
| 25 | 4MEI3M | 4-methoxy-indol-3-ylmethyl | 4-Methoxyglucobrassicin | Indole | C_17_H_22_N_2_O_10_S_2_ | 4.39 | 477.0637 | 96.961 |

^1^ [M-CH_4_OS-H]^-^
