# Supplementary material for: Independent evolution of ancestral and novel defenses in a genus of toxic plants (Erysimum, Brassicaceae)
Source: eLife. 2020 Apr 7;9:e51712. doi: 10.7554/eLife.51712 (PMC7180059; doi:10.7554/eLife.51712)
Supplement: Supplementary file 7. [file elife-51712-supp7.docx]

**Supplementary File 7.** List of candidate cardenolide compounds, determined by exact mass and fragmentation patterns. Asterisks (*) indicate compounds confirmed by commercial standards. Compounds #65 and #78 were excluded due to potential artefact formation with formic acid (see Figure 9-figure supplement 2).

| # | Compound name | Genin | Molecular formula | Retention time [min] | [M+H]^+^ | [M+Na]^+^ | 1^st^ sugar | 2^nd^ sugar | Additional fragments |
| --- | --- | --- | --- | --- | --- | --- | --- | --- | --- |
| 1 | Digitoxigenin* | Digitoxigenin | C_23_H_34_O_4_ | 9.96 | 375.254 | 397.234 | - | - |  |
| 2 |  | Digitoxigenin | C_23_H_34_O_4_ | 10.06 | 375.254 | 397.234 | - |  |  |
| 3 |  | Digitoxigenin | C_29_H_44_O_8_ | 8.81 | 521.312 | 543.293 | Deoxyhexose | - |  |
| 4 |  | Digitoxigenin | C_29_H_44_O_8_ | 9.01 | 521.312 | 543.293 | Deoxyhexose | - |  |
| 5 |  | Digitoxigenin | C_29_H_44_O_8_ | 9.06 | 521.312 | 543.293 | Deoxyhexose | - |  |
| 6 |  | Digitoxigenin | C_29_H_44_O_8_ | 9.12 | 521.312 | 543.293 | Deoxyhexose | - |  |
| 7 |  | Digitoxigenin | C_29_H_44_O_8_ | 9.18 | 521.312 | 543.293 | Deoxyhexose | - |  |
| 8 |  | Digitoxigenin | C_29_H_44_O_8_ | 9.35 | 521.312 | 543.293 | Deoxyhexose | - |  |
| 9 |  | Digitoxigenin | C_29_H_44_O_8_ |  | 521.312 | 543.293 | Deoxyhexose | - |  |
| 10 |  | Digitoxigenin | C_29_H_44_O_9_ | 7.18 | 537.305 | 559.288 | Glucose | - |  |
| 11 |  | Digitoxigenin | C_29_H_44_O_9_ | 7.33 | 537.305 | 559.288 | Glucose | - |  |
| 12 |  | Digitoxigenin | C_29_H_44_O_9_ | 7.76 | 537.305 | 559.288 | Glucose | - |  |
| 13 |  | Digitoxigenin | C_35_H_54_O_13_ | 7.84 | 683.369 | 705.346 | Deoxyhexose | Glucose | 521.311 |
| 14 |  | Digitoxigenin | C_35_H_54_O_13_ | 8.04 | 683.369 | 705.346 | Deoxyhexose | Glucose | 521.311 |
| 15 |  | Digitoxigenin | C_35_H_54_O_13_ | 8.1 | 683.369 | 705.346 | Deoxyhexose | Glucose | 521.311 |
| 16 |  | Digitoxigenin | C_35_H_54_O_13_ | 8.16 | 683.369 | 705.346 | Deoxyhexose | Glucose | 521.311 |
| 17 |  | Digitoxigenin | C_35_H_54_O_13_ | 8.46 | 683.369 | 705.346 | Deoxyhexose | Glucose | 521.311 |
| 18 |  | Digitoxigenin | C_37_H_56_O_14_ | 8.68 | 725.373 | 747.356 | Acetyl-Deoxyhexose | Glucose | 563.322 |
| 19 |  | Digitoxigenin | C_37_H_56_O_14_ | 9.44 | 725.373 | 747.356 | Acetyl-Deoxyhexose | Glucose | 563.322 |
| 20 | Cannogenol | Cannogenol | C_23_H_34_O_5_ | 8.47 | 391.249 | 413.231 | - | - |  |
| 21 |  | Cannogenol | C_29_H_44_O_9_ | 7.02 | 537.304 | 559.288 | Deoxyhexose | - |  |
| 22 |  | Cannogenol | C_29_H_44_O_9_ | 7.43 | 537.304 | 559.288 | Deoxyhexose | - |  |
| 23 |  | Cannogenol | C_29_H_44_O_9_ | 7.7 | 537.304 | 559.288 | Deoxyhexose | - |  |
| 24 |  | Cannogenol | C_29_H_44_O_9_ | 7.78 | 537.304 | 559.288 | Deoxyhexose | - |  |
| 25 |  | Cannogenol | C_29_H_44_O_9_ | 7.98 | 537.304 | 559.288 | Deoxyhexose | - |  |
| 26 |  | Cannogenol | C_29_H_44_O_9_ | 8.04 | 537.304 | 559.288 | Deoxyhexose | - |  |
| 27 |  | Cannogenol | C_31_H_46_O_10_ | 9.02 | 579.319 | 601.3 | Acetyl-Deoxyhexose | - |  |
| 28 |  | Cannogenol | C_35_H_54_O_14_ | 6.82 | 699.359 | 721.341 | Deoxyhexose | Glucose | 537.307 |
| 29 |  | Cannogenol | C_35_H_54_O_14_ | 7.01 | 699.359 | 721.341 | Deoxyhexose | Glucose | 537.307 |
| 30 |  | Cannogenol | C_37_H_56_O_15_ | 7.45 | 741.374 | 763.352 | Acetyl-Deoxyhexose | Glucose | 579.319 |
| 31 |  | Cannogenol | C_37_H_56_O_15_ | 8.06 | 741.374 | 763.352 | Acetyl-Deoxyhexose | Glucose | 579.319 |
| 32 |  | Cannogenin | C_34_H_50_O_12_ | 7.78 | 651.337 | 673.32 | Digitoxose | Xylose | 519.296 |
| 33 |  | Cannogenin | C_35_H_52_O_13_ | 7.34 | 681.348 | 703.33 | Digitoxose | Glucose | 519.296 |
| 34 | Glucocheiranthoside | Cannogenin | C_35_H_52_O_14_ | 6.94 | 697.356 | 719.325 | Deoxyhexose | Glucose | 535.289 |
| 35 |  | *Formyl-cannogenol* | C_30_H_44_O_10_ | 8.01 | 565.302 | 587.283 | Deoxyhexose | - |  |
| 36 |  | *Formyl-cannogenol* | C_30_H_44_O_10_ | 8.18 | 565.302 | 587.283 | Deoxyhexose | - |  |
| 37 |  | *Formyl-cannogenol* | C_36_H_54_O_14_ | 8.37 | 711.360 | 733.342 | Deoxyhexose | Deoxyhexose | 565.302 |
| 38 |  | *Formyl-cannogenol* | C_36_H_54_O_15_ | 7.03 | 727.353 | 749.335 | Deoxyhexose | Glucose | 565.302 |
| 39 |  | *Formyl-cannogenol* | C_36_H_54_O_15_ | 7.15 | 727.353 | 749.335 | Deoxyhexose | Glucose | 565.302 |
| 40 |  | *Acetyl-cannogenol* | C_37_H_56_O_15_ | 7.16 | 741.374 | 763.352 | Deoxyhexose | Glucose | 579.317 |
| 41 |  | *Acetyl-cannogenol* | C_37_H_56_O_15_ | 7.34 | 741.374 | 763.352 | Deoxyhexose | Glucose | 579.317 |
| 42 |  | *Acetyl-cannogenol* | C_37_H_56_O_15_ | 7.41 | 741.374 | 763.352 | Deoxyhexose | Glucose | 579.317 |
| 43 |  | *Acetyl-cannogenol* | C_37_H_56_O_15_ | 7.59 | 741.374 | 763.352 | Deoxyhexose | Glucose | 579.317 |
| 44 |  | Strophanthidin | C_29_H_42_O_9_ | 7.32 | 535.289 | 557.273 | Digitoxose | - |  |
| 45 |  | Strophanthidin | C_29_H_42_O_9_ | 7.4 | 535.289 | 557.273 | Digitoxose | - |  |
| 46 | Helveticoside* | Strophanthidin | C_29_H_42_O_9_ | 7.71 | 535.289 | 557.273 | Digitoxose | - |  |
| 47 |  | Strophanthidin | C_29_H_42_O_10_ | 6.96 | 551.286 | 573.267 | Deoxyhexose | - |  |
| 48 |  | Strophanthidin | C_29_H_42_O_10_ | 7.07 | 551.286 | 573.267 | Deoxyhexose | - |  |
| 49 |  | Strophanthidin | C_29_H_42_O_10_ | 7.29 | 551.286 | 573.267 | Deoxyhexose | - |  |
| 50 |  | Strophanthidin | C_29_H_42_O_10_ | 7.67 | 551.286 | 573.267 | Deoxyhexose | - |  |
| 51 |  | Strophanthidin | C_31_H_44_O_10_ | 8.91 | 577.301 | 599.283 | Acetyl-Digitoxose | - |  |
| 52 |  | Strophanthidin | C_31_H_44_O_10_ | 9 | 577.301 | 599.283 | Acetyl-Digitoxose | - |  |
| 53 |  | Strophanthidin | C_31_H_44_O_11_ | 8.08 | 593.296 | 615.277 | Acetyl-Deoxyhexose | - |  |
| 54 |  | Strophanthidin | C_31_H_44_O_11_ | 8.41 | 593.296 | 615.277 | Acetyl-Deoxyhexose | - |  |
| 55 | Erychroside | Strophanthidin | C_34_H_50_O_13_ | 6.8 | 667.333 | 689.315 | Digitoxose | Xylose | 535.291 |
| 56 |  | Strophanthidin | C_34_H_50_O_14_ | 6.45 | 683.328 | 705.311 | Deoxyhexose | Xylose | 551.271 |
| 57 |  | Strophanthidin | C_35_H_52_O_13_ | 7.14 | 681.348 | 703.33 | Digitoxose | Deoxyhexose | 535.291 |
| 58 | Erysimoside* | Strophanthidin | C_35_H_52_O_14_ | 6.48 | 697.344 | 719.325 | Digitoxose | Glucose | 535.287 |
| 59 |  | Strophanthidin | C_35_H_52_O_14_ | 6.72 | 697.344 | 719.325 | Deoxyhexose | Deoxyhexose | 551.282 |
| 60 | Cheirotoxin | Strophanthidin | C_35_H_52_O_15_ | 6.22 | 713.338 | 735.32 | Gulomethylose | Glucose | 551.286 |
| 61 |  | Strophanthidin | C_36_H_52_O_14_ | 7.48 | 709.344 | 731.326 | Acetyl-Digitoxose | Xylose | 577.301 |
| 62 |  | Strophanthidin | C_36_H_52_O_14_ | 7.89 | 709.344 | 731.326 | Acetyl-Digitoxose | Xylose | 577.301 |
| 63 |  | Strophanthidin | C_37_H_54_O_15_ | 7.12 | 739.365 | 761.336 | Acetyl-Digitoxose | Glucose | 577.301 |
| 64 |  | Strophanthidin | C_37_H_54_O_15_ | 7.15 | 739.365 | 761.336 | Acetyl-Digitoxose | Glucose | 577.301 |
| 65 |  | Strophanthidin | C_37_H_52_O_16_ | 7.51 | 753.331 | 775.316 | Digitoxose | C_8_H_12_O_8_ | 535.289 |
| 66 |  | *Formyl-nigrescigenin* | C_30_H_42_O_11_ | 6.49 | 579.28 | 601.263 | Digitoxose | - |  |
| 67 |  | *Formyl-nigrescigenin* | C_30_H_42_O_11_ | 6.99 | 579.28 | 601.263 | Digitoxose | - |  |
| 68 |  | *Formyl-nigrescigenin* | C_30_H_42_O_12_ | 6.14 | 595.275 | 617.257 | Deoxyhexose | - |  |
| 69 |  | *Formyl-nigrescigenin* | C_30_H_42_O_12_ | 6.21 | 595.275 | 617.257 | Deoxyhexose | - |  |
| 70 |  | *Formyl-nigrescigenin* | C_30_H_42_O_12_ | 6.56 | 595.275 | 617.257 | Deoxyhexose | - |  |
| 71 |  | *Formyl-nigrescigenin* | C_30_H_42_O_13_ | 4.8 | 611.271 | 633.252 | Glucose | - |  |
| 72 |  | *Formyl-nigrescigenin* | C_32_H_44_O_12_ | 8.01 | 621.291 | 643.278 | Acetyl-Digitoxose | - |  |
| 73 |  | *Formyl-nigrescigenin* | C_35_H_50_O_15_ | 6.27 | 711.323 | 733.305 | Digitoxose | Xylose | 579.282 |
| 74 |  | *Formyl-nigrescigenin* | C_35_H_50_O_16_ | 5.88 | 727.317 | 749.299 | Deoxyhexose | Xylose | 595.275 |
| 75 |  | *Formyl-nigrescigenin* | C_36_H_52_O_15_ | 6.62 | 725.338 | 747.32 | Digitoxose | Deoxyhexose | 579.282 |
| 76 |  | *Formyl-nigrescigenin* | C_36_H_52_O_16_ | 5.67 | 741.333 | 763.315 | Digitoxose | Glucose | 579.282 |
| 77 |  | *Formyl-nigrescigenin* | C_36_H_52_O_16_ | 5.95 | 741.333 | 763.315 | Digitoxose | Glucose | 579.282 |
| 78* |  | *Formyl-nigrescigenin* | C_38_H_52_O_18_ | 7.01 | 797.323 | 819.306 | Digitoxose | C_8_H_12_O_8_ | 579.282 |
| 79 |  | Bipindogenin | C_29_H_44_O_10_ | 4.59 | 553.302 | 575.282 | Deoxyhexose | - |  |
| 80 |  | Bipindogenin | C_29_H_44_O_10_ | 4.83 | 553.302 | 575.282 | Deoxyhexose | - |  |
| 81 |  | Bipindogenin | C_29_H_44_O_10_ | 4.9 | 553.302 | 575.282 | Deoxyhexose | - |  |
| 82 |  | Bipindogenin | C_29_H_44_O_10_ | 5.76 | 553.302 | 575.282 | Deoxyhexose | - |  |
| 83 |  | Bipindogenin | C_29_H_44_O_10_ | 5.98 | 553.302 | 575.282 | Deoxyhexose | - |  |
| 84 |  | Bipindogenin | C_29_H_44_O_10_ | 6.64 | 553.302 | 575.282 | Deoxyhexose | - |  |
| 85 |  | Bipindogenin | C_31_H_46_O_11_ | 5.53 | 595.312 | 617.296 | Acetyl-Deoxyhexose | - |  |
| 86 |  | Bipindogenin | C_31_H_46_O_11_ | 6.28 | 595.312 | 617.296 | Acetyl-Deoxyhexose | - |  |
| 87 |  | Bipindogenin | C_35_H_54_O_15_ | 5.22 | 715.365 | 737.346 | Deoxyhexose | Glucose | 553.301 |
| 88 | Nigrescigenin digitoxoside | Nigrescigenin | C_29_H_42_O_10_ | 5.05 | 551.288 | 573.27 | Digitoxose | - |  |
| 89 |  | Nigrescigenin | C_29_H_42_O_11_ | 4.73 | 567.278 | 589.259 | Deoxyhexose | - |  |
| 90 |  | Nigrescigenin | C_29_H_42_O_11_ | 6.8 | 567.278 | 589.259 | Deoxyhexose | - |  |
| 91 |  | Nigrescigenin | C_29_H_42_O_11_ | 6.99 | 567.278 | 589.259 | Deoxyhexose | - |  |
| 92 |  | Nigrescigenin | C_31_H_44_O_12_ | 7.78 | 609.292 | 631.271 | Acetyl-Deoxyhexose | - |  |
| 93 |  | Nigrescigenin | C_31_H_44_O_12_ | 8.23 | 609.292 | 631.271 | Acetyl-Deoxyhexose | - |  |
| 94 |  | Nigrescigenin | C_35_H_52_O_13_ | 4.69 | 683.329 | 705.311 | Digitoxose | Xylose | 551.284 |
| 95 |  | Nigrescigenin | C_35_H_52_O_14_ | 4.92 | 697.344 | 719.324 | Digitoxose | C_6_H_12_O_5_ | 551.281 |
| 96 |  | Nigrescigenin | C_35_H_52_O_15_ | 6.29 | 713.338 | 735.32 | Digitoxose | Glucose | 551.298 |
| 97 | Glucocanescein | Nigrescigenin | C_35_H_52_O_16_ | 6.1 | 729.333 | 751.316 | Gulomethylose | Glucose | 567.284 |
